# Model-Based Comprehensive Analysis of School Closure Policies for Mitigating Influenza Epidemics and Pandemics

**DOI:** 10.1371/journal.pcbi.1004681

**Published:** 2016-01-21

**Authors:** Laura Fumanelli, Marco Ajelli, Stefano Merler, Neil M. Ferguson, Simon Cauchemez

**Affiliations:** 1 Bruno Kessler Foundation, Trento, Italy; 2 MRC Centre for Outbreak Analysis and Modelling, School of Public Health, Imperial College London, London, United Kingdom; 3 Mathematical Modelling of Infectious Diseases Unit, Institut Pasteur, Paris, France; The Pennsylvania State University, UNITED STATES

## Abstract

School closure policies are among the non-pharmaceutical measures taken into consideration to mitigate influenza epidemics and pandemics spread. However, a systematic review of the effectiveness of alternative closure policies has yet to emerge. Here we perform a model-based analysis of four types of school closure, ranging from the nationwide closure of all schools at the same time to reactive gradual closure, starting from class-by-class, then grades and finally the whole school. We consider policies based on triggers that are feasible to monitor, such as school absenteeism and national ILI surveillance system. We found that, under specific constraints on the average number of weeks lost per student, reactive school-by-school, gradual, and county-wide closure give comparable outcomes in terms of optimal infection attack rate reduction, peak incidence reduction or peak delay. Optimal implementations generally require short closures of one week each; this duration is long enough to break the transmission chain without leading to unnecessarily long periods of class interruption. Moreover, we found that gradual and county closures may be slightly more easily applicable in practice as they are less sensitive to the value of the excess absenteeism threshold triggering the start of the intervention. These findings suggest that policy makers could consider school closure policies more diffusely as response strategy to influenza epidemics and pandemics, and the fact that some countries already have some experience of gradual or regional closures for seasonal influenza outbreaks demonstrates that logistic and feasibility challenges of school closure strategies can be to some extent overcome.

## Author Summary

School closure is a non-pharmaceutical intervention, considered for the mitigation of influenza epidemics and pandemics, whose impact and cost-effectiveness are still disputed. We propose the first systematic computational model study assessing the potential impact of different school closure policies. We consider four closure strategies based on school absenteeism: nationwide, countywide, reactive school-by-school, reactive gradual. Mitigation outcomes are highly variable, but under the constraint of a fixed number of weeks lost per student, the optimal implementations of all strategies give comparable results. Gradual and county closures are less sensitive to variations in school absenteeism and thus are slightly more feasible in practice. Our results suggest that health care policy makers may consider school closure as a countermeasure during influenza outbreaks, if put in the condition to properly evaluate the severity of the disease and the subsequent level of effort that a country should be ready to afford.

## Introduction

The identification of effective interventions to mitigate the impact of influenza epidemics is still a public health priority, given that seasonal influenza affects a considerable number of individuals every year, with substantial medical and socio-economic consequences. In addition, it has become even more decisive in recent years in the light of the threat represented by novel influenza viruses, such as H5N1 or H7N9, which might evolve to transmit efficiently in humans [1–4]. School closure is one of the possible non-pharmaceutical measures to hinder an influenza epidemic. This terminology refers in general to unscheduled suspension of school attendance, which can be put into practice in several ways: from class dismissal (lessons are suspended but school remains open with staff) to school closure (school is closed so both students and staff stay home), from proactive (preventive closure in presence of some alert in the community) to reactive closure (when a certain number of students of the same school are sick).

This type of social distancing intervention, which is currently adopted as a countermeasure for seasonal influenza in some countries, was used during the 1918 pandemic in the United States [5,6]. It was also considered as an option in recent pandemic preparedness plans developed by the World Health Organization [7,8], especially during the initial phase of spread, when pharmaceutical interventions (e.g., vaccination) may not be available yet. However, during the 2009 H1N1 influenza pandemic, the WHO did not provide specific recommendations and left any decision on closing schools to national or local authorities [9,10], thus a variety of policies (either favorable or against school closure) and implementations of the measure was applied around the world [11–18].

Some contribution to the control of epidemics is ascribed to school closure since usually school-age children are among the most affected categories of the population, amplifying infection in the entire community [19], and tend to have higher contact rates [20,21]. Moreover, there is evidence that regular school holidays may contribute to reducing transmission of influenza [22–30]. For instance, for the 2009 A/H1N1 pandemic there is evidence suggesting that regular school holidays in the United States resulted in a marked drop in the reproductive number [31] and school reopening dates were a predictor of the timing of the epidemic peak [32]; moreover the only European country showing a major epidemic wave in early summer was the United Kingdom, which was also the only country where schools were still open at the time [28].

However, the cost-effectiveness of school closure as a mitigation measure by itself is still debated [33–35], in particular for the significant socio-economic implications that it brings [36,37], concerning for instance the induced absenteeism of workers that have to look after children or the negative impact on children’s education. For these reasons, its adoption is by no means universal.

Reactive closure of schools with high pupils absenteeism rates has been implemented during seasonal epidemics in Bulgaria since the 1970s [18]; this policy was recommended in a number of countries during the 2009 H1N1 pandemic [18]. Japan and Russia also have a tradition of school closure policies for seasonal influenza outbreaks and typically adopt a gradual closure strategy [18,38], where affected classes close first, then grades and, finally, the entire school. For example, classes where a certain proportion of children are absent due to infection get closed; if at least two classes in a grade are closed, all classes in the grade close; if at least two grades are closed, the whole school gets closed. Quite interestingly, to date this type of school closure has rarely been evaluated by mathematical models assessing mitigation interventions [22,30,33,39–53]. The expected lower societal impact of gradual closure offers an interesting alternative to more intensive closure strategies, as it tackles the major issue of socio-economic costs. However, it presents additional logistic challenges and may fail to have any impact on transmission if surveillance in schools is not sensitive and fast enough.

Another approach can be represented by localized closure of all schools at regional level (e.g. in a county or district), a measure sometimes applied during seasonal influenza outbreaks [18]. Nationwide closure of all schools is rarely considered due to its low sustainability but may be imposed proactively in some situations, as for example in Bulgaria and Serbia during the 2009 influenza pandemic [18].

Here, we use a simulation model to assess the potential impact of these closure interventions in the United Kingdom. Specifically, we compare four different strategies: i) national closure of all schools after a national trigger is passed (hereafter called “national closure”); ii) closure of all schools in a county where a school has been closed reactively (“county closure”); iii) reactive school-by-school closure (“reactive closure”); iv) reactive gradual closure, class-by-class with closure of grades where two or more classes are closed (“gradual closure”). The national closure of all schools is triggered when the cumulative incidence is larger than a certain threshold, which can be based on the national surveillance system. For all other strategies, we consider a protocol defining the surveillance threshold to be reached, which is based on monitoring student excess absenteeism; this seems to be the most realistic option given logistical constraints. Indeed, school closure based on the rapid individuation of confirmed influenza cases could be more effective but very difficult to apply in practice and the effects of unnecessary closure due to false positives should be taken into account as well.

The analysis is performed by making use of an individual based model, structurally similar to that employed in [28,54] and refined to account for a detailed school structure.

## Results

We considered as reference scenario a situation comparable to the experience of the United Kingdom during the 2009 A/H1N1 influenza pandemic: we therefore assumed a basic reproductive number R_0_ = 1.5 (see [55] for a systematic review), probability of developing symptoms given infection was set to 30%, in line with findings reported in [56], and adults were assumed to be half as susceptible to infection as children [33,57,58]. Other assumptions are explained in the Methods section and in the Supporting Text. In the absence of any intervention, the final infection attack rate was estimated to be 19.5% on average (95%CI: 19.4–19.5), similarly to an estimate for the attack rate after the second wave [59], with a mean peak incidence of 6.8 (95%CI: 5.8–7.1) cases per 1,000 individuals, and peak occurring 13.8 weeks on average (95%CI: 12.1–17.2) after the first national case.

**Fig 1:**
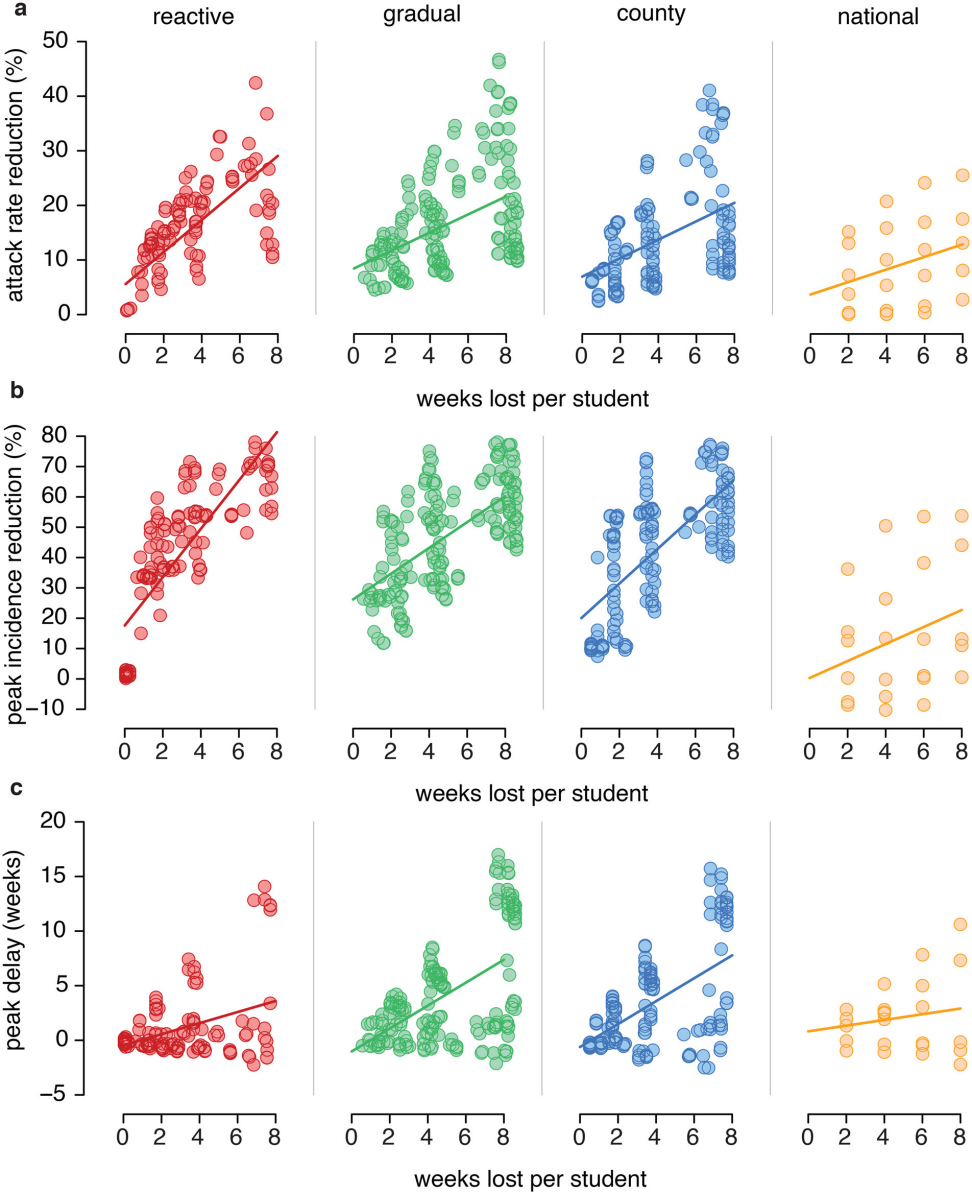
Summary of outcomes of all closure implementations tested. Impact of all closure implementations on key epidemiological indicators, as a function of socio-economic and educational cost (as measured in terms of number of weeks lost). Red: reactive closure, green: gradual closure, blue: county closure, yellow: national closure. Every point represents an implementation of closure with specific duration of single closure events, timeout between consecutive closure events, absenteeism threshold trigger. Lines represent the linear models best fitting simulated data. **a.** Attack rate reduction. **b.** Peak incidence reduction. **c.** Peak delay.

We investigated the effect that different closure strategies (reactive, gradual, county and national) would have on the dynamics of the pandemic in the United Kingdom, with an extensive analysis on the implementation characteristics of closures, namely the duration of each closure event, the timeout between two consecutive closures and the fraction of absent students used as a threshold for triggering closure. Fig 1 presents the impact of each simulated closure strategy on the infection attack rate, the peak incidence, the peak delay and the average number of weeks lost per student: in correspondence to a number of weeks lost spanning from around 0 to 8, the considered school closure implementations yield large variability in attack rate reduction (0–45%, Fig 1a) peak incidence reduction (-10-80%, Fig 1b) and peak delay (-2-18 weeks, Fig 1c) with respect to the case of no intervention. As expected, there is a strong correlation between the economic, social and educational cost of the closure strategy, as measured in terms of weeks lost, and the scale of the reduction for key epidemiological indicators: Spearman correlation test is statistically significant (*p*-value <0.0001) for reactive, gradual and county closures.

In a pandemic context, policy makers would have to carefully weigh the health benefits of school closure against its costs. The level of effort a country will be ready to consent to mitigate a pandemic is a political decision dependent on factors such as the severity of the pandemic or the local public health culture and expectations regarding school closure. An elevated number of weeks lost per student may be acceptable in a severe pandemic but less so in a mild pandemic.

We therefore define the “theoretical” number of weeks lost per student as the maximum level of effort in terms of weeks lost per student, depending on the duration decided for the single closure events. For instance, if the target is an effort of 4 weeks lost, this may correspond to four closures of one week each, two closures of two weeks each, or one closure lasting four weeks; of course, since closures are allowed only within a certain period established by public health authorities (180 days, see Methods), if large threshold trigger and large timeout between consecutive closures are applied, then the single school/class may close for a shorter total period.

**Fig 2:**
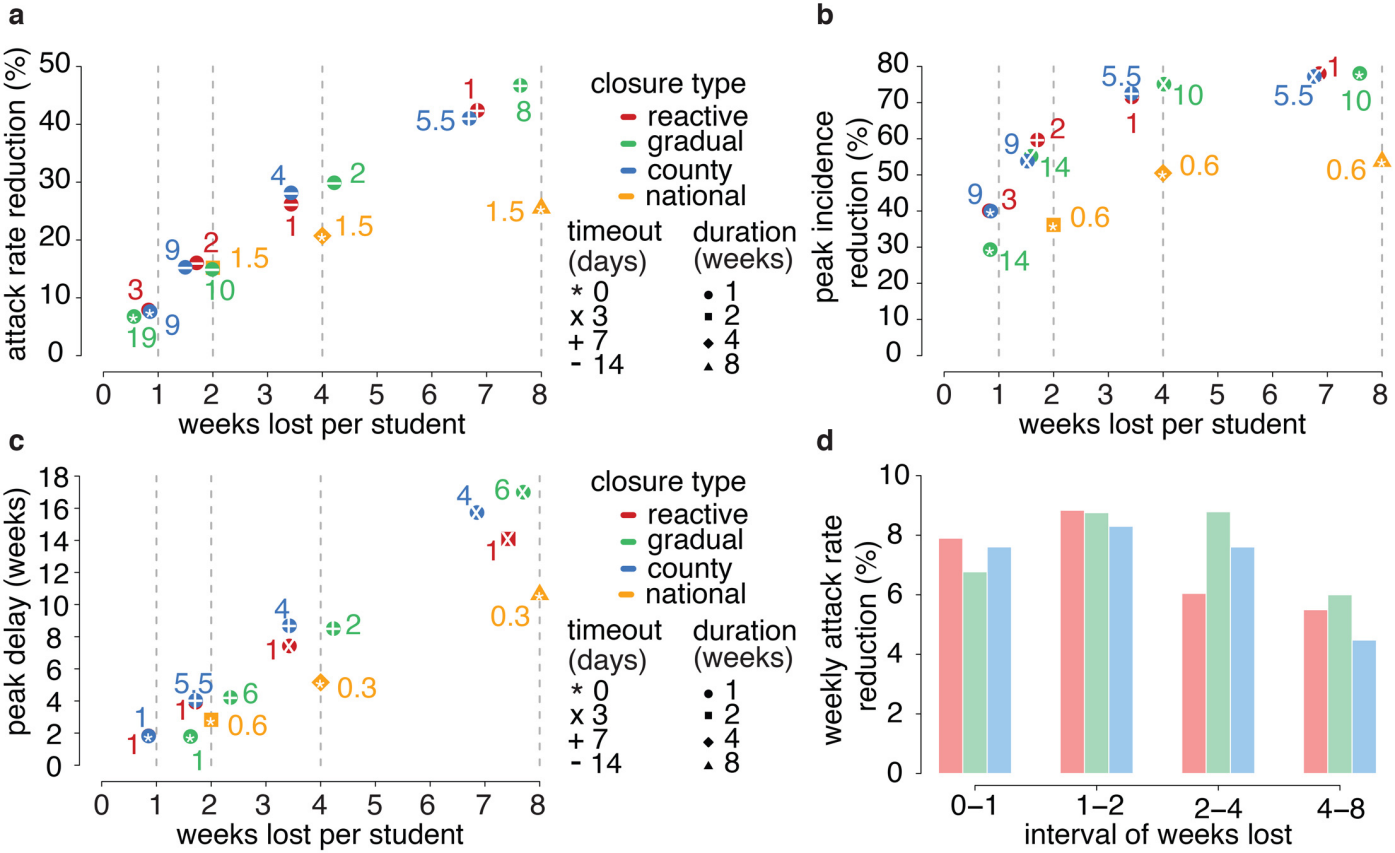
Optimal results as a function of the number of weeks lost per student that policy makers might be ready to consent to mitigate the pandemic. **a.** Closure configurations, among those considered, yielding the optimal attack rate reductions at the end of the policy implementation period, provided that the average theoretical number of weeks lost per student is 1, 2, 4 or 8. Color represents closure type, dot shape represents duration of each single closure event, and white symbol inside a dot represents length of every timeout (see legend). Colored numbers are the excess absenteeism thresholds for each configuration (note that thresholds refer to schools for reactive and county closure, to classes for gradual closure and to cumulative number of symptomatic cases for national closure). **b.** As in **a** but for optimal peak incidence reductions. **c.** As in **a** but for optimal peak delay. **d.** Mean weekly attack rate reduction obtained by increasing the effort from 0 to 1 weeks lost, from 1 to 2 weeks lost, from 2 to 4 weeks lost, and from 4 to 8 weeks lost. Implementations considered are those yielding optimal attack rate reductions, as shown in **a**. Color represents closure type (see legend).

Once a decision is made on the maximum level of effort the country is ready to pay to mitigate the epidemic (1, 2, 4 or 8 weeks lost per student on average), Fig 2 illustrates the strategies yielding the best possible impact under that constraint. For a given target on the theoretical number of weeks lost per student, reactive, gradual and county strategies have similar impact.

In more detail, in a situation where a minimal effort is allowed (i.e., one week lost) closures can cause about 5–10% attack rate reduction at best, and about 30–40% peak incidence reduction, while if 2 weeks lost per student are considered as affordable, we obtain about 15% attack rate reduction and 55–60% peak incidence reduction (Fig 2a and 2b). When assuming the theoretical number of weeks lost to be 4, we obtain around 25–30% attack rate reduction and 70% peak incidence reduction; finally for 8 weeks lost we get 40–45% attack rate reduction and around 80% peak incidence decrease. Interestingly, all optimal interventions are characterized by short durations of closure of one week (roughly corresponding to the time required to break the transmission chains in schools), mostly non-consecutive but separated by intervals of different duration: for instance, optimal attack rate reduction requires timeouts of 2 weeks between consecutive closures for both 2 and 4 weeks lost, but the thresholds for triggering closures are systematically larger for 2 weeks lost, in order to drastically reduce the number of unnecessary closures. When 8 weeks lost are considered as affordable, the optimal interval for attack rate reduction is one week only, probably because a 2-weeks timeout would not allow implementing all feasible closures within the prescribed period. If the intended goal is to delay the epidemic (Fig 2c), the thresholds for optimal excess absenteeism are systematically lower and the timeouts between consecutive closures shorter than those corresponding to the largest attack rate reductions (Fig 2a). In fact, to slow down the epidemic it is necessary to intervene early, when there are few cases, while, in order to reduce infection attack rate, breaking chains of transmission in schools when the epidemic is widespread is much more effective.

Increasing the theoretical number of weeks lost per student yields diminishing marginal returns. In particular, the mean weekly attack rate reduction obtained by increasing the effort from 4 to 8 weeks lost is sensibly lower than that obtained when switching from 0 to 1, or from 1 to 2 weeks lost (Fig 2d).

Impact is systematically lower for national closure (see Fig 2), with the only exception of 2 weeks closure triggered by a 1.5% cumulative national incidence, which would lead to an attack rate reduction very similar to the optimal configurations of the other strategies. Thus national closure may be considered in general as the least effective option. Moreover, as it would involve students (and consequently their caregivers) and school staff of the entire country all at once, this strategy entails high societal costs, probably affordable in case of severe pandemics only.

**Fig 3:**
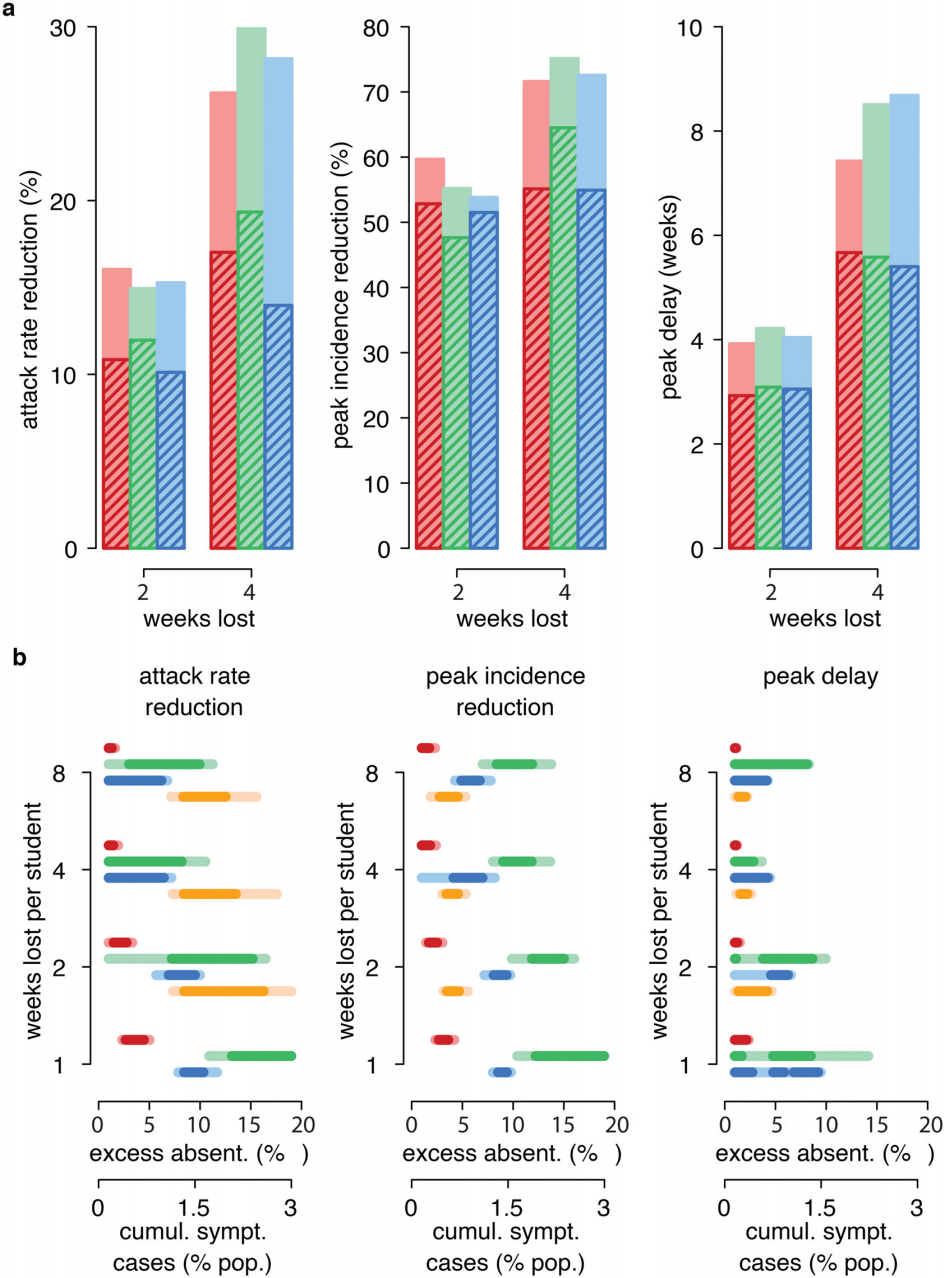
Single closures and sensitivity on excess absenteeism threshold. **a.** Comparison between optimal closures for 2 and 4 weeks lost per student (solid bars) and single closures lasting 2 and 4 weeks yielding the best outcomes in terms of attack rate reduction, peak incidence reduction, or peak delay (shaded bars). Red: reactive closure, green: gradual closure, blue: county closure. **b.** Thresholds of excess absenteeism (for reactive, gradual and county closures–upper x-axis) and of symptomatic cases in the population (for national closure, lower x-axis) yielding attack rate reduction, peak incidence reduction, or peak delay at least 80% (light lines) or 90% (dark lines) of the outcomes obtained by the optimal interventions. Colors as in **a**, yellow represents national closure.

The number of closures for each school/class may be an additional constraint; for instance, based on logistical aspects, policy makers may decide that 2 or 4 weeks lost per student would be affordable but only as a single closure episode. In that case, the corresponding best results would be suboptimal (Fig 3a): attack rate reductions would be about 20% to 33% lower than optimal strategies in the case of 2 weeks lost, and about 35% to 50% lower in the case of 4 weeks lost. Peak would be 1.75 to 3.3 weeks earlier and 4% to 14% larger for 2 weeks lost, around 1 week earlier and 14% to 24% for 4 weeks lost.

Since we ascertained that optimal interventions have similar impact for reactive, gradual and county closure, the point becomes to identify the most feasible, based on the optimal excess absenteeism threshold found: in fact it may be difficult in practice to implement closure according to the precise thresholds identified as optimal, while on the other hand closing when another, possibly similar, threshold is reached may in principle lead to quite different outcomes. To understand this aspect, for all closure types (including national closure) and all expected numbers of weeks lost we considered interventions having the same duration of single closure and the same minimum interval between closures as the optimal configurations. We determined which excess absenteeism thresholds (or cumulative incidence of symptomatic cases in the population for national closure) among those investigated yield attack rate reduction, peak incidence reduction or peak delay no lower than 80% or 90% of the maximum possible. In general, reactive closure is highly sensitive to threshold choice, while gradual closure and county closure are quite stable to small variations in excess absenteeism around the optimal threshold (Fig 3b). This behavior may be motivated with the observation that differences between excess absenteeism thresholds can be remarkable for a school but not for a class. Indeed, in a school of size 300, 1% excess absenteeism–i.e. 7% absent students, whatever the cause–means 21 absent students, 2% means 24 students, 4% means 30 students, 9% means 45 students; but in a class of 25 students, which is the average size in primary schools in the UK [60], 1–2% excess absenteeism–i.e. 7–8% absent students, whatever the cause–means 2 absent students, 6% means 3 students, 10% means 4 students. The county closure approach is much less subject to unnecessary intervention (if the excess absenteeism threshold is reached for a school, causing closure in the entire county, it is likely that at least some other school in the same county is experiencing sickness-induced absenteeism). In summary, these results suggest that gradual and county closures are slightly more feasible in practice.

**Fig 4:**
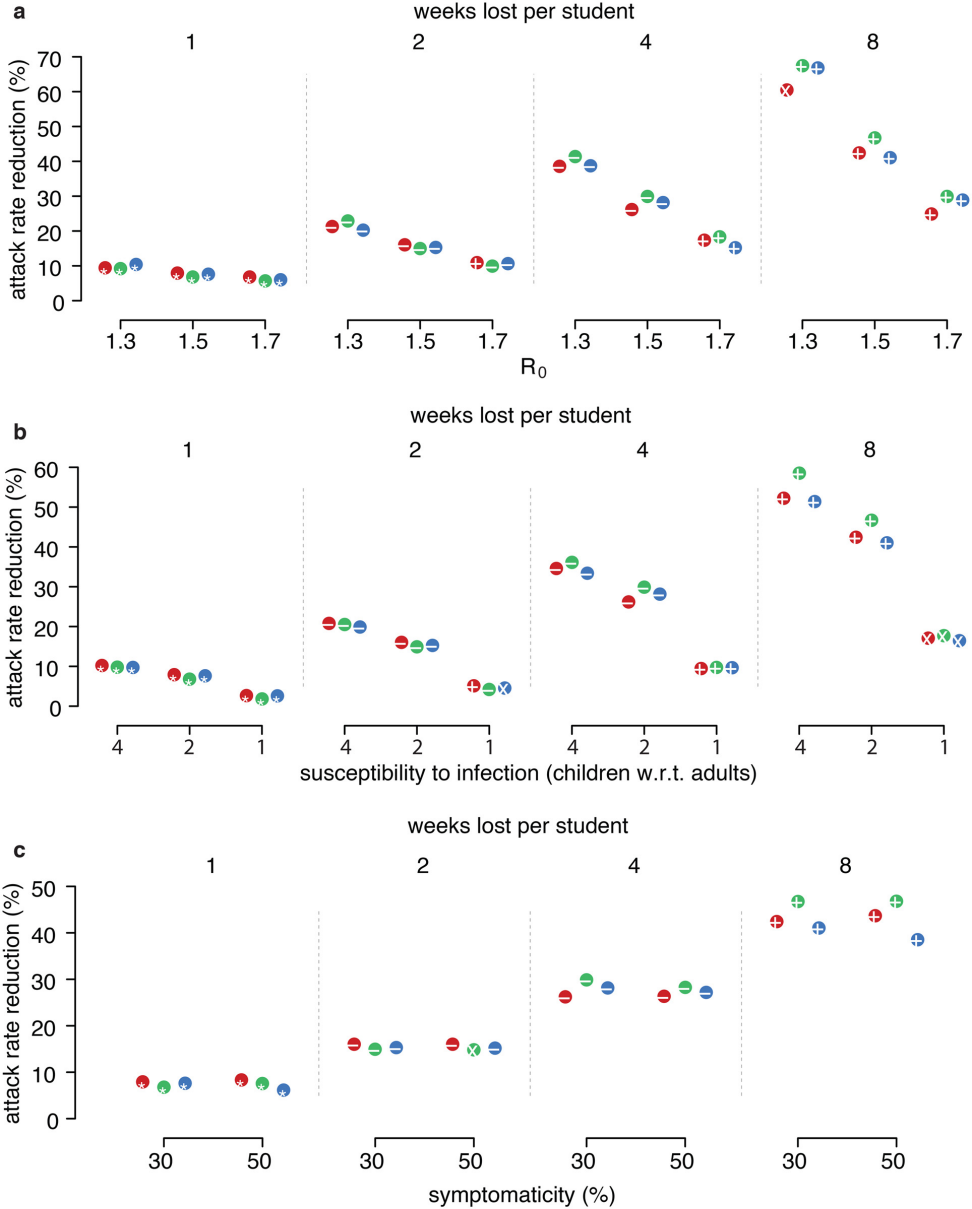
Sensitivity analysis of epidemiological features. Optimal attack rate reductions, depending on the desired average number of weeks lost per student, for different assumptions on the main epidemiological parameters. Colors and symbols as in Fig 1. **a.** Basic reproductive number 1.3, 1.5 and 1.7. **b.** Relative susceptibility of children 4 times that of adults, twice that of adults and same as adults. **c.** Probability of being symptomatic 30% and 50%.

We performed a sensitivity analysis by varying the main epidemiological parameters: we considered R_0_ = 1.3,1.5,1.7; susceptibility to infection of children 4 times higher, twice higher or same as adults; symptomatic cases 30% or 50% of total cases. We let one of the parameters vary while keeping the remaining two fixed. Results for the best attack rate reductions with respect to the case of no intervention are summarized in Fig 4: as expected, mitigation is more effective for lower basic reproductive number and higher susceptibility to infection of children with respect to adults; differences are more evident under the assumption of 4–8 weeks lost per student. Results are more stable with respect to different assumptions on the proportion of symptomatic infections. Overall, there are no substantial differences between the three strategies.

Additional sensitivity analysis is provided in the Supporting Text.

## Discussion

In this study we evaluated alternative options for school closure based on monitoring students’ absenteeism as a potential non-pharmaceutical intervention for the mitigation of influenza pandemic in the United Kingdom.

We found that, under specific constraints on the average number of weeks lost per student, reactive, gradual and county closure gave comparable outcomes in terms of infection attack rate reduction, peak incidence reduction or peak delay, while national closure of all schools of the country at the same time was not able to reach the same levels of mitigation. Optimal implementations generally required short closures of one week each, separated by timeouts of variable duration, so that the chain of transmission inside schools/classes could be broken and unnecessarily prolonged periods of closures avoided; furthermore, we showed that closing each school/class for a single, longer period (2–4 weeks) would be suboptimal.

Results are in qualitative agreement with empirical evidence: for instance national closure (which is quite equivalent to regular holidays since all schools in the country are closed at the same time) causes the value of the reproductive number to drop during the closure period and then increase again at reopening, similarly to what has been observed during the 2009 A/H1N1 influenza pandemic [28,31,32].

Despite being all basically equivalent in terms of mitigation outcomes on equal socio-economic cost (as captured by the average number of school weeks lost per student), gradual and county closures may be slightly more easily applicable in practice than reactive closure, since the corresponding reductions/delays are less sensitive to the choice of the excess absenteeism threshold triggering intervention. Sensitivity analysis on the main epidemiological features of influenza disease confirms these results.

Our aim was to compare different types of reactive school closure with a specific focus on epidemiological aspects, thus we did not perform cost analyses but rather used the average number of school weeks lost per student as a qualitative indicator for the socio-economic cost of each strategy.

One could also attempt to monitor influenza-like illness (ILI) in the school. This choice would be much more challenging since identification of possible ILI cases would imply either contacting parents of absent students to ascertain the reason of their absence, or performing rapid antigen testing of suspected students [14]. However, it would allow more targeted interventions. We did not consider environmental factors (such as absolute humidity), which may play a role in the spread of influenza [61].

Our findings suggest that gradual closure (originating from classes where an excess absenteeism is observed), as well as closure of all schools within the same county of a school where excess absenteeism occurs, may be considered more diffusely by policy makers responding to influenza pandemics, along with reactive and proactive closures that are more typically discussed in pandemic plans. Although possibly slightly more challenging from the operational point of view (since they certainly require an efficient internal surveillance system and a good coordination at regional level respectively), the fact that some countries have already experienced gradual or regional closures for seasonal influenza outbreaks demonstrates that these challenges can be to some extent overcome.

## Methods

### Transmission model

The epidemic model is a spatially explicit, discrete time SEIR model with explicit transmission in households, schools, workplaces and general community, structurally similar to the one developed for Europe in [28,54]. Transmission in schools is further divided into transmission within the class, within the grade (except the class of the infected) and in the school (except the grade of the infected). We assume different infectivity profiles and generation times by age and setting following [62]. Full details on the transmission model are provided in the Supporting Text.

### Model calibration

Parameterization was made starting from empirical data on both past influenza epidemics and the 2009 H1N1pdm pandemic. In particular, transmission rates were estimated for the United Kingdom, in such a way that the following conditions were simultaneously satisfied. First, considering age-dependent susceptibility, with children being twice more susceptible to infection than adults, the overall proportions of cases in the different settings were 27% in households, 40% in schools, 8% in workplaces and 25% in the general community, similarly to [28,58]. Second, the proportion of cases infected in class was the same as those infected in the school, and around 1.35 times those infected in the grade, matching the findings in [62]. Third, we assumed mean generation time to depend on both age and setting: as in [62] we set 3.7 days for students in household, 1.1 days for students at school and in the community, 2.3 days for adults. The corresponding overall effective generation time of the epidemic was 3.2 days, in agreement with [41]. Fourth, the basic reproductive number was set to R_0_ = 1.5 [28].

Different transmissibility scenarios were obtained by multiplying all transmission rates for suitable constant values. For details, see the Supporting Text.

### Closure strategies

We implemented four different closure options:

- reactive: closure of the entire school whenever a certain threshold related to absent students in the school itself is reached;
- gradual: a class is closed whenever a certain threshold related to absent students is reached in the class itself; if two or more classes belonging to the same grade are closed, the whole grade itself gets closed; if two grades are closed, the entire school gets closed;
- county: if a school has been closed reactively, then all schools in the same county get closed;
- national: closure of all schools in the country after a national trigger is passed.

In order to avoid false alarms at the very beginning of the epidemic (when most absenteeism is likely not related to influenza), reactive, gradual and county closures are allowed as soon as the cumulative number of cases experiencing symptoms in the UK as a whole reaches 0.25% of the total population (a sensitivity analysis on this choice is reported in the Supporting Text). Intervention can then be put in place within 180 days since that date. On the other hand, national closure is implemented only once during the course of the influenza season, as soon as a specified (variable) cumulative proportion of symptomatic cases is reached in the total population. In the real world, this would correspond to receiving some alert from the national surveillance system when the epidemic starts being widespread in the country.

The type of trigger considered for all interventions with the exception of national closure is based on the daily monitoring of the number of absent students. We assumed a background–whatever the cause–absenteeism of 6% [63] (i.e. each student has a daily probability 0.06 of not going to school) and that students sick with flu have a certain probability (roughly corresponding to the fraction of influenza cases showing symptoms, thus referred to as ‘symptomaticity’) of staying home for 5 days starting from the day after symptoms onset, thus contributing to increase absenteeism. Closure is triggered by excess absenteeism (i.e. above the background value of 6%) in a school (reactive and county closure)/class (gradual closure) over a specified (variable) threshold. More specifically, the trigger was in the range 1%-9% excess absenteeism of students over the entire school for reactive closure; 1%-19% of students in a class for gradual closure (then a grade was closed when two classes were closed, and the school was closed when two grades were closed); 1%-12% of students of a school for closure of the entire county it belongs to. Reactive, gradual and county closures are then regulated by two other factors: the number of times a school/class may close and the duration of each closure event are expected to be limited; moreover, two consecutive closures will be separated by periods during which schools/classes remain open. Thus we explored different possibilities for the maximum number of closure events (1, 2, 4 times; 8 times in case of 1 week closure), the duration of each closure (1, 2, 4 weeks) and the minimum time interval between two consecutive closures (0, 3, 7 or 14 days).

Concerning national closures, we considered a single closure event lasting 2, 4 or 8 weeks, to be effected when cumulative incidence in the total population is 0.003%-3%.

In the absence of specific information on the behavior of children when classes/schools are closed, we assumed that transmission remained unchanged in households but increased by 25% in the community [22,28,41].

Here we show simulation results right after the 180 days period during which closures are allowed. In the Supporting Text, a comparison with the permanent effect of the different strategies is shown.

## Supporting Information

S1 File Supporting text. Supporting text containing methodological details and additional results.
(PDF)
