# Supplementary material for: Model-Based Comprehensive Analysis of School Closure Policies for Mitigating Influenza Epidemics and Pandemics
Source: PLoS Comput Biol. 2016 Jan 21;12(1):e1004681. doi: 10.1371/journal.pcbi.1004681 (PMC4721867; doi:10.1371/journal.pcbi.1004681)
Supplement: S1 File — Supporting text containing methodological details and additional results. (PDF) [file pcbi.1004681.s001.pdf]

Model-based Comprehensive Analysis of School Closure Policies for Mitigating Influenza Epidemics and Pandemics

Laura Fumanelli[1,*], Marco Ajelli[1], Stefano Merler[1], Neil M. Ferguson[2], Simon Cauchemez[3]

[1] Bruno Kessler Foundation, Trento, Italy

[2] MRC Centre for Outbreak Analysis and Modelling, School of Public Health, Imperial College London, London, UK

[3] Mathematical Modelling of Infectious Diseases Unit, Institut Pasteur, Paris, France

[*] Corresponding author

E-mail: lfumanelli@fbk.eu

Supporting Text

Methods

Model description

We performed our analysis focusing on the United Kingdom by using an individual-based stochastic simulation model similar to the one developed for Europe in [1–3]: highly detailed country-specific socio-demographic data on age structure, household size and composition, employment rates by age, educational system were used to generate a synthetic population where each individual is given an age and is associated to a specific household and an occupation chosen between student, worker and inactive (unemployed/retired). The epidemic model is a spatially explicit, discrete time SEIR model with explicit transmission in households, schools and workplaces and in the general community. Throughout the whole course of the simulation cases from abroad were imported in the UK on the basis of the number of daily airport arrivals from the rest of the world, similarly to [1,4,5].

In the present version, the implemented school organization reflects the real situation existing in the UK in terms of both partitioning of students into different educational levels and class and school size. More in detail, four educational stages have been considered, following the educational structure in England and Wales, and quite in agreement with the situation of Scotland and Northern Ireland: pre-primary school (kindergartens, day-care centers) up to 5 years old, primary school for children from 5 to 11 years old, secondary school from 11 to 18 years old, higher education (post-secondary training, university, doctoral programs) for students older than 18. For each school level the corresponding information on school size has been taken into account in order to obtain a realistic size distribution [6]; in addition, primary and secondary schools have been further partitioned into classes and grades, reflecting information on average size by school level in the UK, namely 25 pupils per class in primary education and 20 students per class in secondary education [7]. All this information have been included in the model in order to build schools of the correct size and to assign every student to the correct school level (from pre-primary to higher education) and grade according to age; moreover, in order to apply mitigation policies based on gradual closure of schools, for primary and secondary schools students belonging to the same school were sub-grouped into classmates, same grade but different class or different grade.

Transmission model

As in [2–4,8] interaction between individuals (and thus infection transmission) can occur in four different settings, namely household, school, workplace or general community. Here we use different, setting-specific infectivity profiles over time and generation times. Moreover, as a consequence of the refinement of primary and secondary schools accounting for classes and grades introduced in our agent based model we were able to split transmission at school into three levels, so that a child can transmit to classmates, to students belonging to the same grade but different class, or to students from other grades, with specific transmission rates as detailed in [9]. In the absence of specific information we assumed transmission rates for class, grade and school to be independent of school level (i.e. primary or secondary education). Since pre-primary schools were not split into classes and grades and their average size was smaller than those of the other

educational levels, the corresponding transmission rate was assumed equal to that in class for the other school levels. The model has therefore six transmission rates as a whole.

As regards infectivity over time, following state-of-the-art estimates on generation time distributions for the 2009 H1N1 influenza pandemic [9], we assumed infectiousness profiles over time to depend on the age of the case and on the setting where interaction occurs. We categorized the population into “students” and “adults” and assumed that interaction would occur in household, at school/workplace and in the general community. We assumed infectivity profiles to be discretized Weibull distributions with an offset of 1 (which is equivalent to having a latency period of 1 day), with parameters depending on whether the case was a student interacting in household, a student interacting outside the household (i.e. at school or in the community), or an adult. At any time t, any infectious individual i can infect others in every setting he belongs to, with probability $p_i(t) = 1 - \exp(-\lambda_i \Delta t)$, where $\lambda_i(t)$ is the force of infection exerted at time t by individual i in that specific setting. For instance, the force of infection of infectious individual i on another member of the same household is

$$\lambda_i(t) = k^H(t - \tau_i; a_i)\beta^H / N_i$$

where $k^H = k^H(t - \tau_i; a_i)$ is the infectivity of the case in the household, which is a function of the time of infection τ_i and of the age a_i of the case, β^H is the transmission rate in the household and N_i is the household size. Analogous expressions describe the force of infection in schools, workplaces and general community. We assumed that, during school closure, household transmission remained unchanged, but community transmission increased of 25% [4].

Natural history of influenza and model calibration

Given our choice to consider infectivity profiles to depend on age (student/adult) and transmission setting, we assumed the value of the mean generation time to be 3.7 days (standard deviation 3.1 days) for students in household; 1.1 days (standard deviation 0.4 days) for students at school and in the community; 2.3 days (standard deviation 2.9 days) for adults [9].

The transmission model was parameterized in such a way that the overall proportions of cases in the different settings, assuming no differential susceptibility between adults and children, were consistent with empirical estimates, namely: 30% in households; 18% in schools; 19% in workplaces; 33% in the general community [4,10–12], while assuming age-dependent susceptibility to infection led to 27% of transmission in households, 40% in schools, 8% in workplaces and 25% in the general community, similarly to what has been found in [2]. In addition, to match findings of [9], the proportion of transmission in class was set to be the same as the proportion of transmission in the entire school, and around 1.35 times the proportion of transmission in the grade. Different transmissibility scenarios were obtained by multiplying all transmission rates for suitable constant values.

Choosing generation time distributions and transmission rates determines the evolution of the epidemic.

Following the argument proposed in [13] the basic reproduction number R_0 is generally defined as the average number of secondary infections caused by a typical primary infection in a fully susceptible population [14]. However, the definition of a typical infectious individual for a model with highly heterogeneous mixing such as the one used in this work is not straightforward. Therefore, in the parameterization procedure we considered in place of R_0 the quantity R_{rand}, which is defined as the average number of secondary infections generated by a randomly selected individual in a fully susceptible population [4]. Ferguson et al. [4] showed that for US and UK populations the relation $R_0 = R_{rand} + 0.2$ holds. In our simulations yielding $R_{rand} = 1.5$ we estimate ad epidemic growth rate of 0.221 days^{-1}, in excellent agreement with the estimate of 0.219 days^{-1} in [4] for simulations having $R_{rand} = 1.5$ and $R_0 = 1.7$. This allows presenting all results in terms of R_0 rather than R_{rand}, making it easier to compare them with the ones reported in the literature.

For classic homogeneous mixing SIR models, it is well known [4,15] that $R_0 = 1 + rT_g$, where T_g is the overall generation time and r is the exponential growth rate of the epidemic. Since we are considering setting and age-specific generation time distributions, we do not know the value of the overall generation time for our model; moreover, we want to test whether this approximation still holds given the high heterogeneity of our model and, if so, for which value of the generation time (named T_{eff}). By minimizing the quantity

$$f(T_{eff}) = \sum_{j=R_0\in\{1.1,\dots,2.3\}} \left(\frac{\frac{1}{r_j} - \frac{T_{eff}}{j-1}}{\frac{1}{r_j}} \right)^2$$

where r_j is the growth rate of the simulated epidemic having $R_0 = j$, we obtain $T_{eff} = 3.2$ days, in perfect agreement with the findings reported in [4] and we can conclude that the relation between R_0 and T_g is a good approximation in the explored range $R_0 = 1.1, \dots, 2.3$. This justifies the choice, already used for instance in [2,8], of multiplying transmission rates for suitable scaling factors in order to obtain different transmissibility scenarios.

Basic reproductive number

The transmission model was calibrated assuming a basic reproductive number R_0=1.5, to be consistent with estimates for the 2009 H1N1 influenza pandemic presented in [16]. R_0 was computed from the intrinsic growth rate during the initial phase of the simulated epidemic without assuming any kind of intervention, initialized with 100 infectious individuals randomly chosen in the population so that stochastic effects were partially eliminated. As a sensitivity analysis we also explored scenarios with R_0=1.3, 1.7.

Probability of being symptomatic

In the reference scenario we considered the proportion of influenza cases showing symptoms to be 30%, as described in [17]. 50% probability of being symptomatic was also explored as sensitivity analysis.

Differential susceptibility by age

In the reference scenario we assumed children to be twice more susceptible to infection than adults, as estimated for the 2009 A/H1N1 influenza pandemic as well as for other influenza epidemics [9,18,19]; in the main text we also explored the scenarios where children are either as susceptible or four times more susceptible than adults.

Time of simulation

We set the day when the first case in the population occurred as the initial time of the simulation, and let the simulation run for 600 days. Closure interventions can be implemented as soon as the cumulative number of cases experiencing symptoms in the UK as a whole reaches 0.25% of the total population (corresponding to ~140,000 individuals), and during the following 180 days.

Number of realizations

In order to ensure stability of findings, all presented results were obtained by averaging over 50 stochastic realizations of the same experiment. Around 26,000 simulations were carried out as a whole to produce the full analysis. Variability among simulations was larger for the epidemic timing, due to the stochastic nature of the initial phases, while final infection attack rate, depending on the course of the entire epidemic, was very stable.

Additional results

Start of interventions

The choice, made in the main text, to begin the application of interventions as soon as the cumulative number of cases experiencing symptoms in the UK as a whole reaches 0.25% or higher of the total population is arbitrary. To perform a sensitivity analysis on this threshold, we considered the framework corresponding to the best attack rate reduction provided to lose 4 weeks, and assumed to allow closures after the first case in the population (which represents an extreme situation) or as soon as cumulative symptomatic cases were 0.5% or 1% of the total population. We obtained that, for all closure types, all thresholds on the initial cumulative incidence yield attack rate reduction comparable to the baseline 0.25% threshold, while starting closures after the very first case would be too early, causing too little impact on attack rate (Fig S1). This means that a cumulative case incidence of 0.25% or higher may be a good trigger for starting closures while avoiding false alarms that may not be unusual in the very first phases of the epidemic.

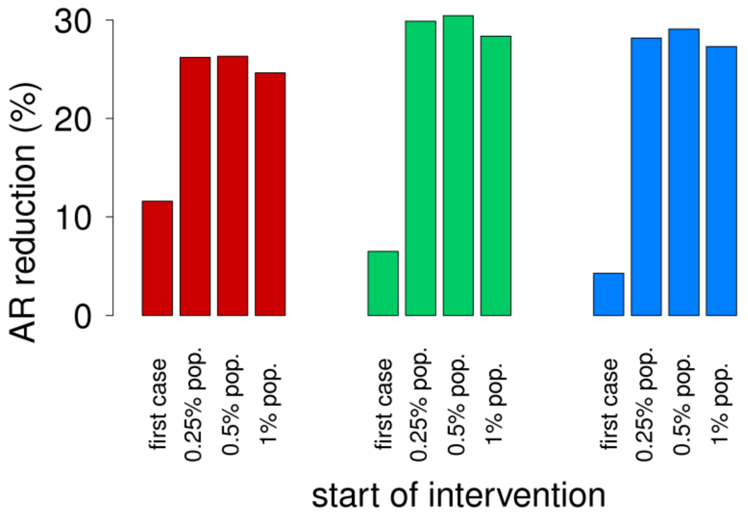

Fig S1. Start of intervention. *Final attack rate reduction for different options on when to allow first implementation of school closure: after the first national case or as soon as 0.25%, 0.5% or 1% of the national population gets infected with symptoms. The considered framework is the one yielding the best final attack rate reduction when 4 weeks lost per student are allowed. Red bars: reactive closure; green bars: gradual closure; blue bars: county closure.*

Transient and permanent effects

All results presented in the main text are evaluated right after the end of the period during which application of closure policies is possible: we may refer to this as the "transient" behavior of the epidemic. Of course, in principle the epidemic may not be over by that time and a resurgence of cases would still be possible, so we analyze the main epidemiological indicators (i.e. attack rate reduction, peak incidence reduction, peak delay) of the best strategies also at the end of the simulation. We assumed the final time of each simulation to be 600 days after the first national case, without considering holidays typically planned during the school year in order to emphasize the effect of unscheduled closures. We infer (Fig S2) that the only situation where some significant difference between transient and final behavior can be observed is attack rate reduction for 8 weeks lost: in that case best reactive, gradual and county closures yield a 40-45% attack rate reduction with respect to the baseline scenario at the end of closure implementation, dropping to about 30% at the end of the simulation. The only other marginal difference between transient and final attack rate reduction can be observed for county closure at 4 weeks lost. This means that all best implementations (for whatever indicator and closure type), applicable in a period lasting 180 days, are almost always able to halt the epidemic, with the only relevant exception of 8 weeks lost, where a second wave of cases at the end of the closure implementation period can be observed (see Fig S3).

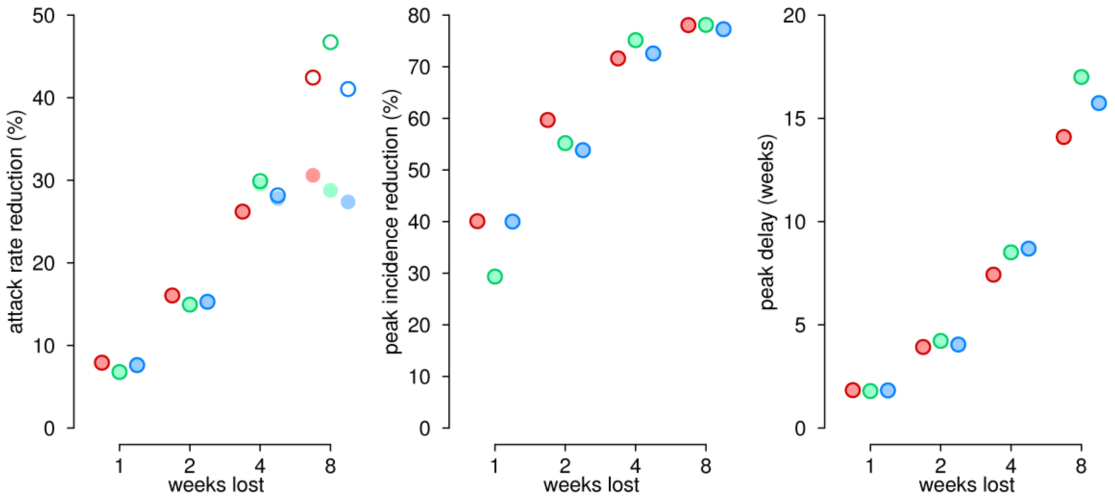

Fig S2. Transient vs. permanent effects. *Relevant indicators of the mitigated epidemics resulting from the optimal strategies evaluated right at the end of the closure implementation period (empty circles) and at the final time of the simulation (solid circles), in terms of the number of weeks lost per student. Red: reactive closure; green: gradual closure; blue: county closure.*

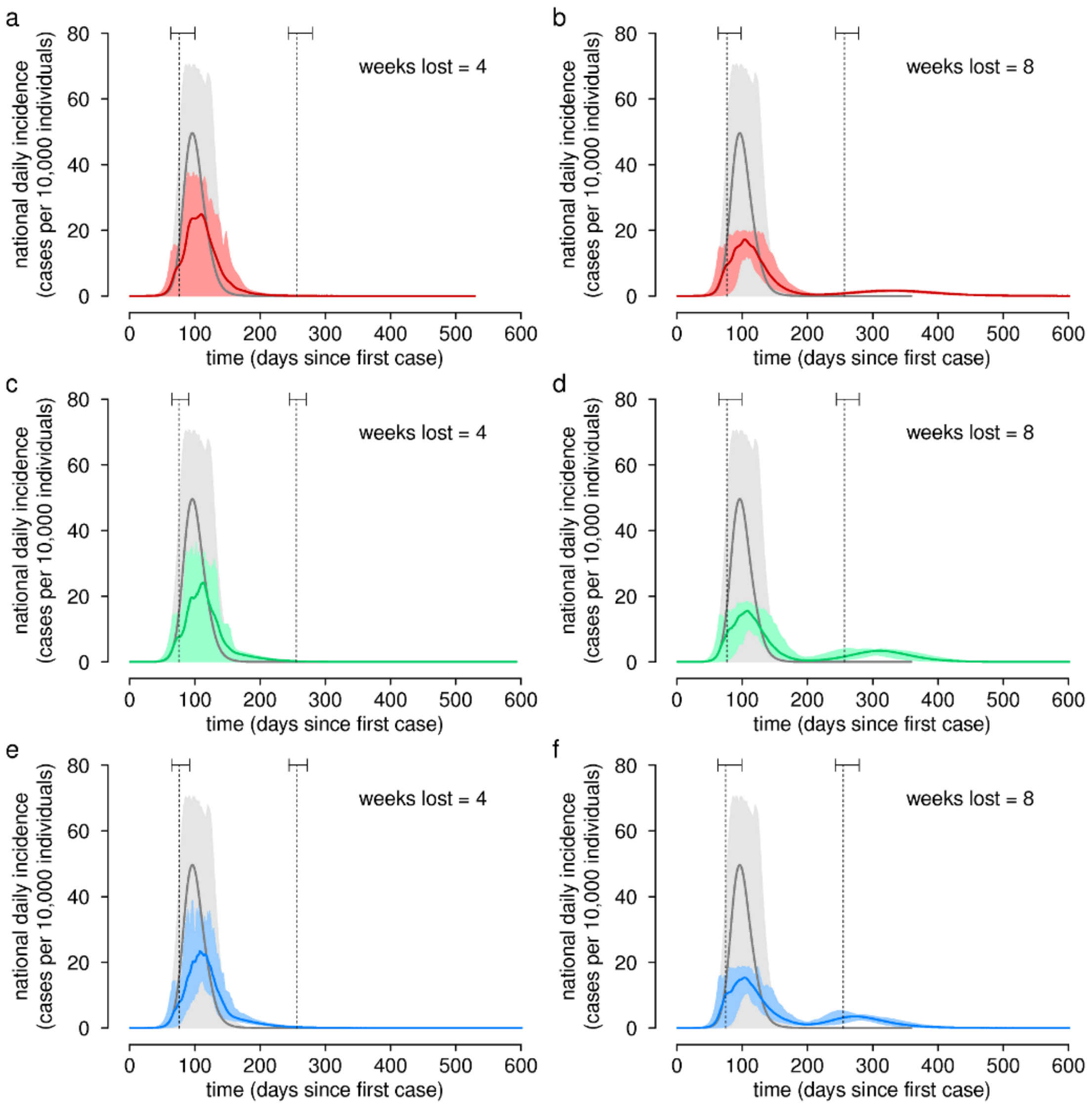

Fig S3. Daily incidence. *National daily cases per 10,000 individuals for the epidemic without any closure intervention (grey) and epidemic resulting from optimal closure strategies in terms of transient attack rate reduction (red: reactive closure; green: gradual closure; blue: county closure). Solid lines represent mean values from the total simulation runs; shaded regions represent the corresponding 95% confidence intervals. Vertical lines correspond to the starting and ending dates of the period during which closures can be implemented, with their 95% confidence intervals represented on top.* ***a, c, e.*** *Four weeks lost per student.* ***b, d, f.*** *Eight weeks lost per student.*

References

1. Merler S, Ajelli M. The role of population heterogeneity and human mobility in the spread of pandemic influenza. Proc R Soc B Biol Sci. 2010;277(1681):557–65.

2. Merler S, Ajelli M, Pugliese A, Ferguson NM. Determinants of the Spatiotemporal Dynamics of the 2009 H1N1 Pandemic in Europe: Implications for Real-Time Modelling. PLoS Comput Biol. 2011;7(9):e1002205.

3. Fumanelli L, Ajelli M, Manfredi P, Vespignani A, Merler S. Inferring the Structure of Social Contacts from Demographic Data in the Analysis of Infectious Diseases Spread. PLoS Comput Biol. 2012;8(9):e1002673.

4. Ferguson NM, Cummings DAT, Fraser C, Cajka JC, Cooley PC, Burke DS. Strategies for mitigating an influenza pandemic. Nature. 2006;442(7101):448–52.

5. Ciofi degli Atti ML, Merler S, Rizzo C, Ajelli M, Massari M, Manfredi P, et al. Mitigation Measures for Pandemic Influenza in Italy: An Individual Based Model Considering Different Scenarios. PLoS ONE. 2008;3(3):e1790.

6. Education, Audiovisual and Culture Executive Agency, European Commission. Key data on education in Europe 2009. 2009. Available from: http://eacea.ec.europa.eu/education/eurydice/documents/key_data_series/105en.pdf

7. Statistical Office of the European Commission. Pupil/student-teacher ratio and average class size (ISCED 1-3). 2012. Available from: http://appsso.eurostat.ec.europa.eu/nui/show.do?dataset=educ_iste&lang=en

8. Ferguson NM, Cummings DAT, Cauchemez S, Fraser C, Riley S, Meeyai A, et al. Strategies for containing an emerging influenza pandemic in Southeast Asia. Nature. 2005;437(7056):209–14.

9. Cauchemez S, Bhattarai A, Marchbanks TL, Fagan RP, Ostroff S, Ferguson NM, et al. Role of social networks in shaping disease transmission during a community outbreak of 2009 H1N1 pandemic influenza. Proc Natl Acad Sci. 2011;108(7):2825–30.

10. Cauchemez S, Carrat F, Viboud C, Valleron AJ, Boëlle PY. A Bayesian MCMC approach to study transmission of influenza: application to household longitudinal data. Stat Med. 2004;23(22):3469–87.

11. Cauchemez S, Valleron A-J, Boëlle P-Y, Flahault A, Ferguson NM. Estimating the impact of school closure on influenza transmission from Sentinel data. Nature. 2008;452(7188):750–4.

12. Kwok KO, Leung GM, Riley S. Modelling the Proportion of Influenza Infections within Households during Pandemic and Non-Pandemic Years. PLoS ONE. 2011;6(7):e22089.

13. Merler S, Ajelli M, Fumanelli L, Vespignani A. Containing the accidental laboratory escape of potential pandemic influenza viruses. BMC Med. 2013;11(1):252.

14. Anderson RM, May RM. Infectious diseases of humans: dynamics and control. Oxford Univ. Press; 1992.

15. Wallinga J, Lipsitch M. How generation intervals shape the relationship between growth rates and reproductive numbers. Proc R Soc Lond B Biol Sci. 2007;274(1609):599–604.

16. Biggerstaff M, Cauchemez S, Reed C, Gambhir M, Finelli L. Estimates of the reproduction number for seasonal, pandemic, and zoonotic influenza: a systematic review of the literature. BMC Infect Dis. 2014;14(1):480.

17. Hayward AC, Fragaszy EB, Bermingham A, Wang L, Copas A, Edmunds WJ, et al. Comparative community burden and severity of seasonal and pandemic influenza: results of the Flu Watch cohort study. Lancet Respir Med. 2014;2(6):445–54.

18. Fraser C, Donnelly CA, Cauchemez S, Hanage WP, Van Kerkhove MD, Hollingsworth TD, et al. Pandemic Potential of a Strain of Influenza A (H1N1): Early Findings. Science. 2009;324(5934):1557–61.

19. Cauchemez S, Donnelly CA, Reed C, Ghani AC, Fraser C, Kent CK, et al. Household Transmission of 2009 Pandemic Influenza A (H1N1) Virus in the United States. N Engl J Med. 2009;361(27):2619–27.
